# Melatonin modulates red-ox state and decreases viability of rat pancreatic stellate cells

**DOI:** 10.1038/s41598-020-63433-6

**Published:** 2020-04-14

**Authors:** Antonio Gonzalez, Matias Estaras, Salome Martinez-Morcillo, Remigio Martinez, Alfredo García, Mario Estévez, Patricia Santofimia-Castaño, Jose A. Tapia, Noelia Moreno, Marcos Pérez-López, María P. Míguez, Gerardo Blanco-Fernández, Diego Lopez-Guerra, Miguel Fernandez-Bermejo, Jose M. Mateos, Daniel Vara, Vicente Roncero, Gines M. Salido

**Affiliations:** 1 Institute of Molecular Pathology Biomarkers, University of Extremadura, Caceres, Spain; 2 Unit of Toxicology, Veterinary Faculty, University of Extremadura, Caceres, Spain; 3 Department of Animal Health, Veterinary Faculty, University of Extremadura, Caceres, Spain; 4 Department of Animal Production, CICYTEX-La Orden, Guadajira, Badajoz, Spain; 5 IPROCAR Research Institute, Food Technology, University of Extremadura, 10003 Cáceres, Spain; 6 Centre de Recherche en Cancérologie de Marseille, INSERM U1068, CNRS UMR 7258, Aix-Marseille Université and Institut Paoli-Calmettes, Parc Scientifique et Technologique de Luminy, Marseille, France; 7 Hepatobiliary-Pancreatic Surgery and Liver Transplant Unit, Infanta Cristina Hospital, Badajoz, Spain; 8 Department of Gastroenterology, San Pedro de Alcantara Hospital, Caceres, Spain; 9 Unit of Histology and Pathological Anatomy, Veterinary Faculty, University of Extremadura, Caceres, Spain

**Keywords:** Cancer, Cell biology, Cell growth

## Abstract

In this work we have studied the effects of pharmacological concentrations of melatonin (1 µM–1 mM) on pancreatic stellate cells (PSC). Cell viability was analyzed by AlamarBlue test. Production of reactive oxygen species (ROS) was monitored following CM-H_2_DCFDA and MitoSOX Red-derived fluorescence. Total protein carbonyls and lipid peroxidation were analyzed by HPLC and spectrophotometric methods respectively. Mitochondrial membrane potential (ψ_m_) was monitored by TMRM-derived fluorescence. Reduced (GSH) and oxidized (GSSG) levels of glutathione were determined by fluorescence techniques. Quantitative reverse transcription-polymerase chain reaction was employed to detect the expression of Nrf2-regulated antioxidant enzymes. Determination of SOD activity and total antioxidant capacity (TAC) were carried out by colorimetric methods, whereas expression of SOD was analyzed by Western blotting and RT-qPCR. The results show that melatonin decreased PSC viability in a concentration-dependent manner. Melatonin evoked a concentration-dependent increase in ROS production in the mitochondria and in the cytosol. Oxidation of proteins was detected in the presence of melatonin, whereas lipids oxidation was not observed. Depolarization of ψ_m_ was noted with 1 mM melatonin. A decrease in the GSH/GSSG ratio was observed, that depended on the concentration of melatonin used. A concentration-dependent increase in the expression of the antioxidant enzymes catalytic subunit of glutamate-cysteine ligase, catalase, NAD(P)H-quinone oxidoreductase 1 and heme oxygenase-1 was detected in cells incubated with melatonin. Finally, decreases in the expression and in the activity of superoxide dismutase were observed. We conclude that pharmacological concentrations melatonin modify the redox state of PSC, which might decrease cellular viability.

## Introduction

It is nowadays increasing the focus of research on the role of pancreatic stellate cells (PSC) in the physiology and the pathophysiology of the pancreas. PSC comprise of a rather small cell population of the organ. Under normal conditions PSC remain quiescent, but become activated in disease. Activated PSC are responsible for the progressive fibrosis and for the accumulation of extracellular matrix that occurs in severe pancreatic disorders such as chronic pancreatitis and pancreatic cancer^1,2^. Therefore, it is thought that activated PSC are involved in tumor progression and chemoresistance. In this regard, PSC contribute to stromal or fibrotic reaction by the release of matrix components, release signaling molecules that act on neighboring cells to modulate their proliferation and tissue growth within cancer^3^. Unraveling the mechanisms underlying growth and proliferation of PSC is of major relevance for the understanding of pancreatic diseases. In this line, it is tempting to find drugs whose anti-inflammatory, anti-fibrotic and/or anti-proliferative actions could be used in therapy.

Melatonin (N-acetyl-5-methoxytryptamine) is a compound that is produced mainly, but not exclusively, in the pineal gland. Initially, it was considered a hormone with key roles in the regulation of circadian rhythms, conveying physiological and neuroendocrine functions within the body. However, melatonin is also produced in other parts of the organism, as for example retina, Harderian gland, gastrointestinal tract, testes and lymphocytes where it can induce local effects^4^. The compound exerts its actions acting through its specific receptors or directly. Melatonin can bind to cellular membrane MT1- and MT2-type receptors, or can interact with intracellular proteins, as for example nuclear receptor ROR/RZR, quinone reductase 2 (termed MT3 type receptor) and calmodulin^5–8^. Beside its actions as a circadian regulator, especially of reproduction, melatonin also works as free radical scavenger, through potentiation of antioxidant defenses or via immune modulation, thereby exerting protective roles on cell physiology^8^. On the contrary, melatonin also induces cell death^8,9^. Interestingly, all these effects are cell- and context-dependent^8^. With time, widespread attention on the effects of melatonin on cellular physiology and, especially, on its ability to control cell proliferation in cancer has emerged. Melatonin induces antitumor effects in different tissues^10–13^, including the pancreas^14,15^. The anticarcinogenic effects of melatonin involve different mechanisms, as for example apoptosis and cancer immunity. In addition, melatonin diminishes autophagy, metastasis and angiogenesis, leading in general to a decrease of proliferation of malignant cells^16^.

As mentioned above, PSC depict an important role as components of the tumor microenvironment and have emerged as key modulators in the context of tissue injury. In this regard, we have shown that melatonin modulates proliferation of murine^17^ and human PSC^18^. Our previous results showed that melatonin induced Ca^2+^ mobilization from intracellular pools and activation of key components of the mitogen-activated protein kinases (MAPKs) family. In addition, in human PSC a decrease in the GSH/GSSG ratio was observed, which could compromise cellular antioxidant defenses and induce prooxidant conditions that could diminish cell survival. Therefore, melatonin might be a compound with putative parallel effects on the cells forming part of a growing tumor, controlling their proliferation.

In the present study we aimed at identifying new actions of melatonin on the pancreas which might highlight the compound as potential candidate in therapy. We have continued our former studies to further investigate the ways by which melatonin could exert its effects on PSC to control their proliferation.

## Materials and Methods

### Pancreatic tissues and chemicals

Pancreatic tissues used in this study were obtained from newborn *Wistar* rats (one week). Animals employed have been purchased from the animal house of the University of Extremadura (Caceres, Spain). Animals handling, methods and experimental protocols were approved by, and were carried out according to, the University Ethical Committee (reference 57/2016) and by the Institutional Committee of the Junta de Extremadura (reference 20160915). Additionally, all methods and the experimental protocols were performed in accordance with the relevant guidelines and regulations of the Ethical Committee for Animal Research of the University of Extremadura and with the Institutional Committee of the Junta de Extremadura (law 32/2007 and RD 53/2013).

Most chemicals and reagents used for the present work were purchased from Sigma-Aldrich (Merck, Madrid, Spain) and AbD serotec (BioNova Científica, Madrid, Spain). The enzyme collagenase CLSPA for digestion of the pancreas was purchased from Worthington Biochemical Corporation (Labclinics, Madrid, Spain). The components for the preparation of culture medium and the fluorescent probes used were obtained from Invitrogen (Fisher Scientific Inc., Madrid, Spain) and from BioWhittaker (Lonza, Basel, Switzerland). Plastic materials for cell culture were purchased from Thermo Fisher Sci. (Madrid, Spain). Materials and reagents for Western blotting were purchased from Bio-Rad (Madrid, Spain) and from Cell Signaling Technology (C-Viral, Madrid, Spain). Superoxide dismutase (SOD) activity, total antioxidant capacity (TAC) kits were purchased from BioVision (Deltaclon S.L., Madrid, Spain).

The antibodies and primers used were purchased from Thermo Scientific (Fisher Scientific Inc., Madrid, Spain), Sigma-Aldrich (Merck, Madrid, Spain) and Santa Cruz Biotechnologies Inc. (Quimigen S.L., Madrid, Spain).

### Pancreatic stellate cells cultures

PSC were prepared and cultured using established methods^17^. After preparation of cells suspension, small aliquots were seeded on polystyrene plates for cell culture. Culture medium consisted of medium 199, plus 4% horse serum, 10% FBS, 0.1 mg/mL streptomycin, 100 IU penicillin and 1 mM NaHCO_3_. The cells were grown under constant temperature (37 °C) and CO_2_ (5%). Confluence (90–95%) was reached after eight-ten days of culture.

### Study of cell viability

Cells were treated with different stimuli for 48 h. Determination of cell viability was carried out according to previous techniques^19^. A plate reader was used to monitor absorbance (VariosKan Lux 3020–205, Thermo Sci., Vantaa, Finland). The viability of cells subjected to stimuli was compared with that of control cells (non-stimulated). Data show the change in cell viability expressed as the mean in percentage ± S.E.M. (n) with respect to non-stimulated cells (n is the number of experiments carried out).

### Detection of reactive oxygen species (ROS) generation

ROS generation was monitored employing methods used in or laboratory^20^. Cells were detached and loaded with CM-H_2_DCFDA (10 µM) or with MitoSOX Red (2.5 µM). Next, cells were incubated with stimuli during 1 h. For detection of changes in the red-ox state cells were excited at 530 nm and fluorescence emitted was detected at 590 nm for CM-H_2_DCFDA, whereas for cells loaded with MitoSOX red excitation at 510 nm with detection at 580 were employed. A spectrofluorimeter was used to monitor fluorescence (VariosKan Lux 3020–205, Thermo Sci., Vantaa, Finland). Results show the mean increase of fluorescence expressed in percentage ± SEM (n) with respect to non-stimulated cells, where n is the number of independent experiments, as described previously^20^.

### Detection of protein Carbonyls (Allysine)

Cells were incubated during 1 h with stimuli and, thereafter, were lysed for analysis. Detection of protein carbonyls was performed according to the methods described by Villaverde *et al*.^21^. In brief, five hundred µL of each sample were treated with cold 10% trichloroacetic acid (TCA) solution. After centrifugation (600 × *g* for 5 min at 4 °C) the supernatants were removed and the pellets were sequentially incubated with a solution containing 0.5 mL 250 mM 2-(N-morpholino) ethanesulfonic acid (MES) buffer pH 6.0 containing 1 mM diethylenetriaminepentaacetic acid (DTPA), a solution containing 0.5 mL 50 mM ABA in 250 mM MES buffer pH 6.0 and a solution containing 0.25 mL 100 mM NaBH_3_CN in 250 mM MES buffer pH 6.0. Next, samples were treated with a cold 50% TCA solution and centrifuged at (1200 × g for 10 min). The pellets were then washed twice with 10% TCA and diethyl ether-ethanol (1:1). Finally, the pellet was treated with 6 M HCl and kept in an oven at 110 °C for 18 h until completion of hydrolysis. Thereafter, the samples were dried *in vacuo* and the generated residue was reconstituted with 200 µL of milliQ water and filtered for HPLC analysis using a Shimadzu ‘Prominence’ HPLC apparatus (Shimadzu Corporation, Japan). The elutes were monitored with excitation and emission wavelengths set at 283 and 350 nm, respectively. Standards (0.1 μL) were run and analysed under the same conditions. The nmol of allysine per mg of protein were calculated. Results are expressed as percentage ± SEM (n) with respect to non-stimulated cells, where n is the number of independent experiments.

### Analysis of thiobarbituric-reactive substances

Cells were incubated during 1 h with stimuli and, thereafter, were lysed for analysis. Malondialdehyde (MDA) and other thiobarbituric-reactive substances (TBARS) were measured, by adding 500 µL thiobarbituric acid (0.02 M) and 500 µL trichloroacetic acid (10%) to 200 µL of a sample from each treatment. Next, the mixture was incubated for 20 min at 90 °C. After cooling, a 5 min centrifugation at 600 × *g* was made and the absorbance of supernatant was measured at 532 nm employing a plate reader (VariosKan Lux 3020–205, Thermo Sci., Vantaa, Finland). The mg/L of TBARS in each sample were calculated. Results are expressed as percentage ± SEM (n) with respect to non-stimulated cells, where n is the number of independent experiments.

### Determination of mitochondrial membrane potential

Changes in mitochondrial membrane potential (ψ_m_) were recorded using the dye TMRM as described previously^22^. Cells were incubated during 1 h in the presence of stimuli. A decrease in TMRM fluorescence reflects depolarization of ψ_m_. Fluorescence was measured employing a spectrofluorimeter (VariosKan Lux 3020–205, Thermo Sci., Vantaa, Finland). The experiments were carried out employing batches of cells obtained from different preparations. The increase of fluorescence with respect to non-stimulated cells was calculated and expressed in percentage as the mean ± SEM (n) (n is the number of experiments).

### Determination of glutathione levels

The changes in the levels of reduced (GSH) and oxidized (GSSG) glutathione were determined using methods described previously^18^. Cells were incubated during 4 h with the different stimuli assayed. A spectrofluorimeter (Tecan Infinite M200, Grödig, Austria) was employed to detect GSH or GSSG at 350 nm/420 nm (excitation/emission) respectively. For quantification, standard curves of GSH and GSSG were used. Normalization was carried out based on the total protein concentration in each sample^23^. A standard curve was prepared using bovine serum albumin. The experiments were carried out employing batches of cells obtained from different preparations.

Data are shown as the mean increase in GSH/GSSG ratio expressed in percentage ± SEM (n) with respect to non-stimulated cells, where n is the number of independent experiments.

### Quantitative reverse transcription-polymerase chain reaction (RT-qPCR) analysis

This procedure was carried out as previously described^24^. PSC in culture were incubated during 4 h with different stimuli and lysed. Total RNA samples were purified using a commercially available kit (Sigma, Madrid, Spain). The Power SYBR Green RNA-to-C_T_ 1-Step kit (Applied Biosystems, Township, USA) was used. Reverse transcription was performed for 30 min at 48 °C, and PCR conditions were 10 min at 95 °C followed by 40 cycles of 15 s at 95 °C plus 1 min at 55 °C using the following primers:

*Gclc*:5′-GGCACAAGGACGTGCTCAAGT-3′ and 5′-TGCAGAGTTTCAAGAACATCG-3′

*Cat*:5′-ACTTTGAGGTCACCCACGAT-3′ and 5′-AACGGCAATAGGGGTCCTCTT-3′

*Ho-1*:5′-AGCACAGGGTGACAGAAGAG-3′ and 5′-GAGGGACTCTGGTCTTTGTG-3′

*Nqo-1*:5′-GGGGACATGAACGTCATTCTCT-3′ and 5′-AAGACCTGGAAGCCACAGAAGC-3′

*Gapdh*:5′-GGGTGTGAACCACGAGAAAT-3′ and 5′-CCTTCCACGATGCCAAAGTT-3′

*SOD1*: 5′-GGGGACAATACACAAGGCTGTA-3′ and 5′-CAGGTCTCCAACATGCCTCT-3′

*SOD2*: 5′-GTGGAGAACCCAAAGGAGAG-3′ and 5′-GAACCTTGGACTCCCACAGA-3′

The mRNA abundance of each transcript was normalized to the *Gapdh* mRNA abundance obtained in the same sample. The relative mRNA levels were calculated using the ΔΔCt method, and were expressed as the fold change between sample and calibrator. The experiments were carried out employing batches of cells obtained from different preparations.

### Determination of SOD activity

This procedure was carried out using a commercially available kit from BioVision. Stimuli were added to the cells and were incubated during 1 h. Thereafter SOD activity was determined following the manufacturer’s directions. The sensitive SOD assay kit utilizes WST-1 that produces a water-soluble formazan dye upon reduction with superoxide anion.

The activity of SOD can be determined by a colorimetric method. Absorbance at 450 nm of the samples was measured employing a spectrofluorimeter (VariosKan Lux 3020–205, Thermo Sci., Vantaa, Finland). The experiments were carried out employing batches of cells obtained from different preparations. Results show the mean change of absorbance expressed in percentage ± SEM (n) with respect to non-stimulated cells, where n is the number of independent experiments.

### Determination of total antioxidant capacity

Total antioxidant capacity (TAC) was determined using a commercially available kit from BioVision, following manufacturer’s directions. Absorbance at 570 nm of the sample was measured employing a plate reader (CLARIOstar Plus, BMG Labtech., C-Viral, Madrid, Spain). Results show the mean change of absorbance expressed in percentage ± SEM (n) with respect to non-stimulated cells, where n is the number of independent experiments.

### Western blotting analysis

Western blotting was performed using previously described methods^14^. Cells in culture were incubated in the presence of different stimuli during 1 h and lysed. Bradford’s method was used for quantification of the protein content of lysates^23^. Protein lysates (12 µg/lane) of each sample were separated by SDS-PAGE, using 10% polyacrylamide gels, and were transferred to nitrocellulose membranes. Specific primary and the corresponding IgG-HRP conjugated secondary antibody were used for detection of proteins. Quantification of the intensity of the bands which appear was performed using the software *ImageJ* (http://imagej.nih.gov/ij/). The experiments were carried out employing batches of cells obtained from different preparations. Values are expressed as the mean ± SEM of normalized values expressed as % *vs* control (non-stimulated) cells.

### Statistical analysis

Statistical analysis of data was performed by one-way analysis of variance (ANOVA) followed by Tukey *post hoc* test, and only *P* values < 0.05 were considered statistically significant. For individual comparisons and statistics between individual treatments we employed the Student’s *t* test, and only *P* values <0.05 were considered statistically significant.

## Results

### Effects of melatonin on cell viability

It has been suggested that melatonin modulates cell viability of different cellular types^9,14,25,26^, including PSC^17,18^. At this point it was of interest to corroborate the effect of melatonin on cell viability. Thus, PSC were incubated in the absence (non-treated cells) or in the presence of 1 mM, 100 µM, 10 µM or 1 µM melatonin, and cell viability was evaluated after 48 h of culture. The viability of cells that had been incubated in the presence of melatonin was compared with that of non-treated cells.

**Figure 1:**
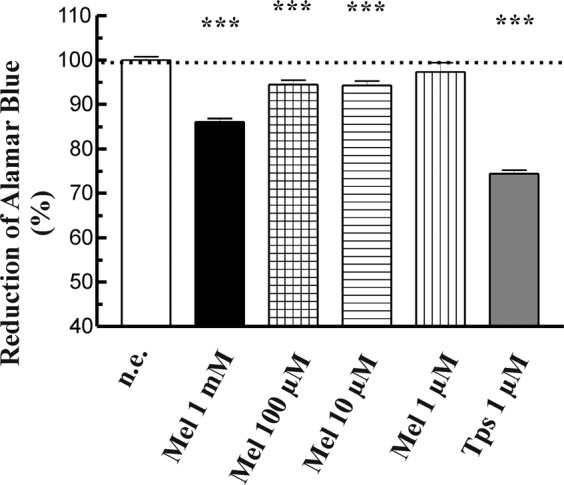
Analysis of PSC viability. Cell viability was analyzed studying AlamarBlue reduction by viable cells. Cells were incubated during 48 h in the presence of melatonin (Mel; 1 mM, 100 µM, 10 µM or 1 µM) or thapsigarging (Tps, 1 µM) and viability was compared with that of cells in the absence of stimulus (control). In the graph, a dotted line represents the viability of control cells (non-treated cells). Histograms are representative of three independent experiments (n.e.,non-stimulated cells; Mel, melatonin; Tps, thapsigargin; ***P < 0.001 *vs* non-stimulated cells).

Cell viability dropped in the presence of 10 µM to 1 mM of melatonin (Fig. 1). A maximal effect was noted with 1 mM melatonin. Separate batches of cells were treated with 1 µM thapsigargin (Tps), which served as control for cell death^27^. In the presence of Tps a strong decrease in cell viability was observed.

### Effect of melatonin on cellular oxidative state

**Figure 2:**
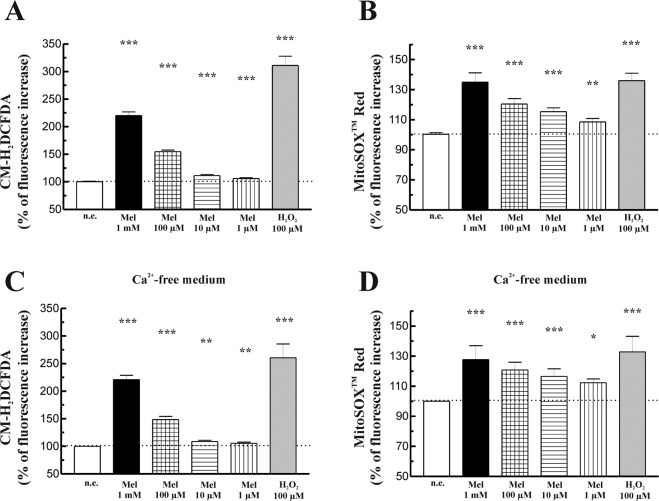
Generation of ROS in response to melatonin. (**A**) Cells were loaded with the red-ox-sensitive dye CM-H_2_DCFDA and were challenged with different concentrations of melatonin (1 mM, 100 µM, 10 µM or 1 µM). As a control, cells were incubated in the presence of 100 µM hydrogen peroxide (H_2_O_2_). (**B**) Cells were loaded with the mitochondrial superoxide indicator MitoSOX Red and were incubated in the presence of melatonin (1 mM, 100 µM, 10 µM or 1 µM). Separated batches of cells were incubated with 100 µM hydrogen peroxide (H_2_O_2_). (**C** and **D**) Cells, loaded with either of the mentioned dyes, were challenged with melatonin in the absence of Ca^2+^ in the extracellular medium (medium containing 0.5 mM EGTA). The bars show the mean increase of dye-derived fluorescence expressed in percentage ± SEM with respect to control (non-stimulated) cells. A horizontal dotted line represents the value observed in non-stimulated cells. Results are representative of six independent experiments (n.e., non-stimulated cells; Mel, melatonin; *P < 0.05; **P < 0.01; ***P < 0.001 *vs* non-stimulated cells).

It has been suggested the melatonin may exert a pro-oxidant action that could underlie its antiproliferative actions^28^. To study this possibility we analyzed the effect of melatonin on ROS production. For this purpose PSC were loaded with the ROS-sensitive fluorescent dyes CM-H_2_DCFDA or MitoSOX Red. Thereafter, cells were incubated during 1 h with melatonin (1 mM, 100 µM, 10 µM or 1 µM). The compound evoked a concentration-dependent increase in ROS production both in the cytosol and in the mitochondria. Hydrogen peroxide (100 µM) was used as a control of oxidation. For this purpose the oxidant was added to the cells, which were then incubated during 1 h. In the presence of hydrogen peroxide a statistically significant increase in dye-derived fluorescence was observed, reflecting an increase in oxidation (Fig. 2A,B).

Increases of cellular calcium (Ca^2+^) have been related with ROS generation and with pancreatic disease^22,29^. In a former work we have shown that melatonin induces mobilization of Ca^2+^ in PSC^17^. In order to check whether ROS generation in response to melatonin was dependent on Ca^2+^, we performed a series of experiments in which PSC were challenged in the absence of extracellular Ca^2+^ (medium containing 0.5 mM EGTA). Under these conditions ROS production evoked by melatonin did not differ from that observed in the presence of Ca^2+^ (Fig. 2C,D).

**Figure 3:**
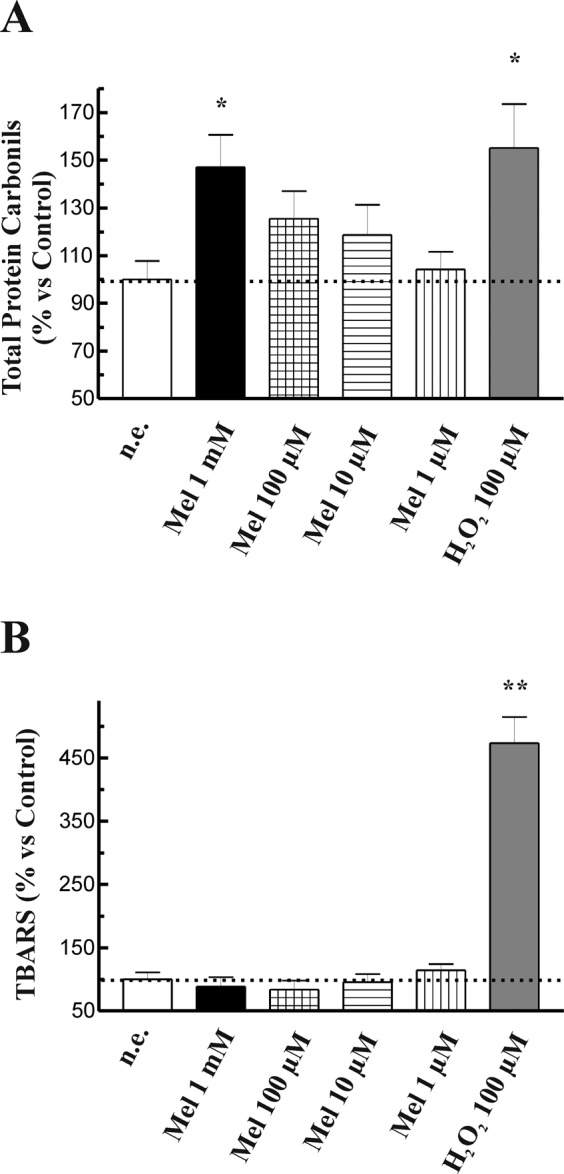
Effect of melatonin on protein and lipid oxidation. PSC were incubated during 1 h in the presence of melatonin (1 mM, 100 µM, 10 µM or 1 µM), and the effect on total protein carbonyls (**A**) or TBARS (**B**) were assayed. 100 µM H_2_O_2_ was used as control of oxidation. The bars show the mean change expressed in percentage ± SEM with respect to control (non-stimulated) cells. A horizontal dotted line represents the value observed in non-stimulated cells. Results are representative of six independent experiments (n.e., non-stimulated cells; Mel, melatonin; H_2_O_2_, hydrogen peroxide; *P < 0.05; **P < 0.01 *vs* non-stimulated cells).

In order to investigate whether the increase in ROS production was accompanied by lipid and/or protein oxidation, the effect of melatonin on protein carbonyl levels and on TBARS were assayed. For this purpose, cells were incubated during 1 h in the presence of melatonin (1 mM, 100 µM, 10 µM or 1 µM). H_2_O_2_ (100 µM) was used as control. We observed a concentration-dependent increase in the total protein carbonyls content in cells treated with melatonin in comparison with that noted in non-stimulated cells. A maximal effect was observed in response to 1 mM melatonin (Fig. 3A). However, no statistically significant changes were detected in the levels of TBARS (Fig. 3B). Treatment of cells with H_2_O_2_ (100 µM) induced statistically significant increases in both total protein carbonyls and TBARS (Fig. 3A,B).

### Effect of melatonin on mitochondrial membrane potential

**Figure 4:**
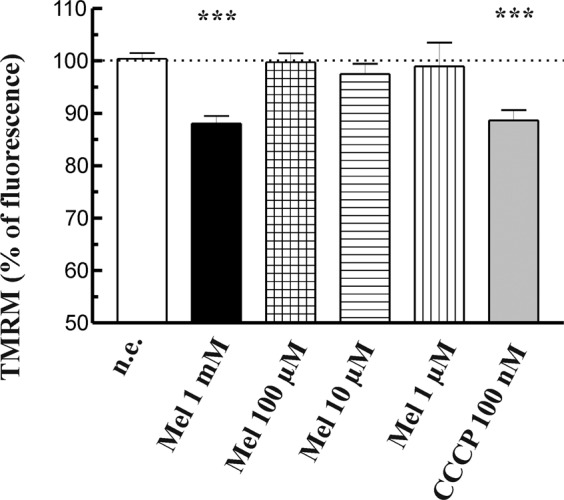
Effect of melatonin on mitochondrial membrane potential. PSC were loaded with the mitochondria-specific voltage-sensitive dye TMRM. The cells were then incubated during 1 h in the presence of melatonin (1 mM, 100 µM, 10 µM or 1 µM). As a control, different batches of cells were incubated in the presence of the mitochondrial uncoupler CCCP (100 nM). The bars show the changes in ψ_m_ of treated and non-stimulated (control) cells, and are presented as the mean increase of fluorescence expressed in percentage ± SEM with respect to non-stimulated cells. A horizontal dotted line represents the value observed in non-stimulated cells (n.e., non-stimulated cells; Mel, melatonin; ***P < 0.001 *vs* non-stimulated cells; n = four independent experiments).

It has been suggested that oxidative stress and changes in ψ_m_ are closely related^30^. In order to analyze whether melatonin induces changes in ψ_m_, we performed a series of experiments in which PSC were loaded with the mitochondria-specific voltage-sensitive dye TMRM. The cells were then incubated during 1 h in the presence of melatonin (1 mM, 100 µM, 10 µM or 1 µM). We could only observe a statistically significant decrease in ψ_m_ in cells treated with 1 mM melatonin. No detectable changes in ψ_m_ were noted in response to the other concentrations of melatonin employed. As a control, different batches of cells were incubated in the presence of the mitochondrial uncoupler CCCP^22,31^. In the presence of CCCP (100 nM) a statistically significant decrease in ψ_m_ was detected (Fig. 4).

### Effect of melatonin on glutathione levels

**Figure 5:**
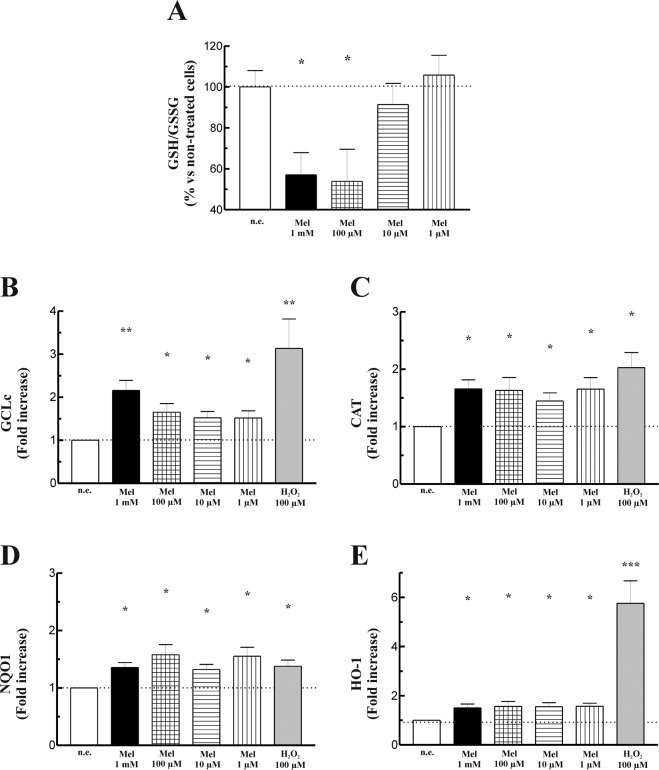
Effect of melatonin on glutathione. (**A**) PSC were incubated during four h in the presence of melatonin (1 mM, 100 µM, 10 µM or 1 µM), and the effect on glutathione was analyzed. The bars show the mean increase in GSH/GSSG ratio expressed in percentage ± SEM with respect to non-stimulated cells. (**B-D**) RT-qPCR analysis of Nrf2-target genes glutamate cysteine ligase-catalytic subunit (GClc), catalase (CAT), NAD(P)H quinone oxidoreductase 1 (NQO1) and heme-oxygenase-1 (HO-1) reveals statistically significant increases in the levels of Nrf2-dependent antioxidant enzymes in cells incubated in the presence of melatonin. Incubation of cells with H_2_O_2_ (100 µM) also evoked an increase in the expression of all four antioxidant enzymes. *Gapdh* mRNA was used for normalization. Data are expressed as the mean ± S.E.M. of the change relative to non-stimulated cells. A horizontal dotted line represents the value observed in non-stimulated cells. Three different cellular preparations were used (n.e., non-stimulated cells; Mel, melatonin; *P < 0.05; **P < 0.01).

Glutathione represents a major antioxidant defense against oxidative stress^32^. Because we had observed ROS production in the presence of the melatonin, it was of interest to test its effect on the glutathione system in PSC. Therefore, cells were incubated during 4 h in the presence of melatonin (1 mM, 100 µM, 10 µM or 1 µM) and the levels of GSH and GSSG were analyzed. We observed a concentration-dependent decrease in GSH/GSSG ratio in cells treated with melatonin in comparison with that noted in non-stimulated cells. A maximal effect was observed in response to 1 mM or 100 µM melatonin. A slight decrease in GSH/GSSG ratio was observed in response to 10 µM melatonin, which was not statistically significant. Whereas we did not detect changes in GSH/GSSG ratio in cells treated with 1 µM melatonin (Fig. 5A).

### Effect of melatonin on Nrf2-dependent antioxidant enzymes

Nrf2 is a transcription factor that enhances the expression of a multitude of antioxidant and phase II enzymes, which regulate redox homeostasis^33^. The results shown above indicate that melatonin induces changes in the redox status of PSC. Therefore, we decided to study whether melatonin could stimulate the transcriptional activation of certain antioxidant enzymes through the activation of Nrf2. For this purpose PSC were incubated during 4 h in the presence of melatonin (1 mM, 100 µM, 10 µM or 1 µM) and RT-qPCR of the relative mRNA abundance was performed. Melatonin evoked statistically significant increases in the expression of GCLc, CAT, NQO1 and HO-1 (Fig. 5B–D). As a control, cells were incubated in the presence of H_2_O_2_ (100 µM), a known Nrf2 activator^34^. The oxidant increased the expression of all four antioxidant enzymes studied.

### Effect of melatonin on superoxide dismutase

**Figure 6:**
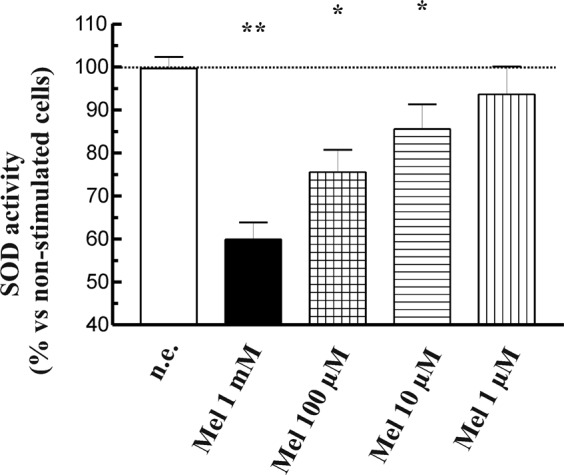
Effect of melatonin on SOD activity. PSC were incubated during 1 h in the presence of melatonin (1 mM, 100 µM, 10 µM or 1 µM). The bars show the mean change of SOD activity expressed in percentage ± SEM with respect to control (non-stimulated) cells. A horizontal dotted line represents the value observed in non-stimulated cells. Results are representative of five independent experiments (n.e., non-stimulated cells; Mel, melatonin; *P < 0.05; **P < 0.01 *vs* non-stimulated cells).

Superoxide dismutases (SOD) catalyze the dismutation of superoxide anion (O2^−^) to H_2_O_2_, which is then catalyzed to innocuous O_2_ and H_2_O by glutathione peroxidase and catalase. Thus, SOD is involved in the defense system against ROS^35^. Several classes of SOD have been identified: Cu/Zn SOD (SOD1), which is localized in cytosol, and MnSOD (SOD2), which is localized in mitochondria^36,37^. We were intereseted in analyzing whether melatonin exerted any affect on SOD. Thus, PSC were incubated during 1 h with the compound (1 mM, 100 µM, 10 µM or 1 µM) and SOD activity was then analyzed. In the presence of melatonin a concentration-dependent decrease in SOD activity was observed (Fig. 6).

**Figure 7:**
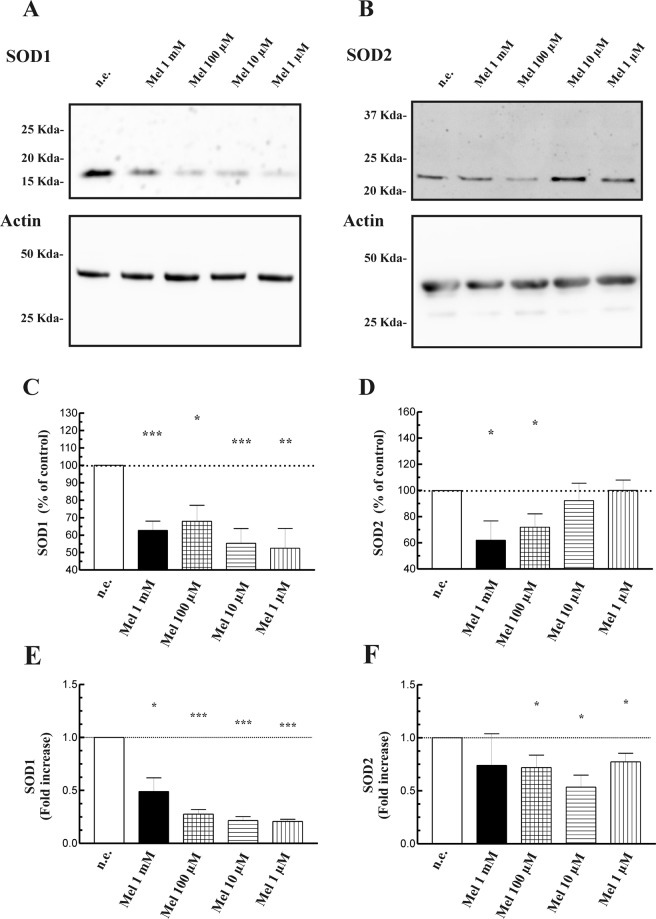
Expression of SOD in PSC treated with melatonin. PSCs were incubated during 1 h in the absence (Control) or in the presence of the desired concentration of melatonin (1 mM, 100 µM, 10 µM or 1 µM). The figure shows representative blots showing the effect of melatonin on the level of the antioxidant enzymes SOD1 (**A**) and SOD2 (**B**), evaluated with specific antibodies. The levels of actin were employed as controls to ensure equal loading of proteins. (**C** and **D**) The graphs show the quantification of protein expression. A horizontal dotted line represents the value observed in non-stimulated cells. Values are the mean ± S.E.M. of normalized values expressed as % of phosphorylation in control (non-stimulated) cells. (**E** and **F**) RT-qPCR analysis was performed to detect mRNA levels of SOD1 and SOD2 respectively. The bars show the mean ± S.E.M. of the change in mRNA levels of each protein relative to non-stimulated cells. *Gapdh* mRNA was used for normalization. A horizontal dotted line represents the value observed in non-stimulated cells. Three different cellular preparations were used (n.e., non-stimulated cells; Mel, melatonin; *P < 0.05; **P < 0.01; ***P < 0.001 *vs* non-stimulated cells). The experiments shown are representative of three different preparations.

We further analyzed the effect of melatonin on SOD and decided to study the protein levels of the enzyme by Western blotting. The results show that PSC that had been incubated with melatonin exhibited lower levels of both SOD1 and SOD2, compared with non-treated cells. The stronger decrease of protein expression was noted for SOD1 (Fig. 7A–D).

Additional studies were carried out to confirm the effect of melatonin on SOD expression. PSC were incubated during 1 h in the presence of melatonin (1 mM, 100 µM, 10 µM or 1 µM) and RT-qPCR of the relative mRNA abundance of SOD1 and SOD2 were performed. In cells treated with melatonin, statistically significant decreases in the mRNA of both proteins were observed (Fig. 7E–F).

### Effect of melatonin on the total antioxidant capacity

**Figure 8:**
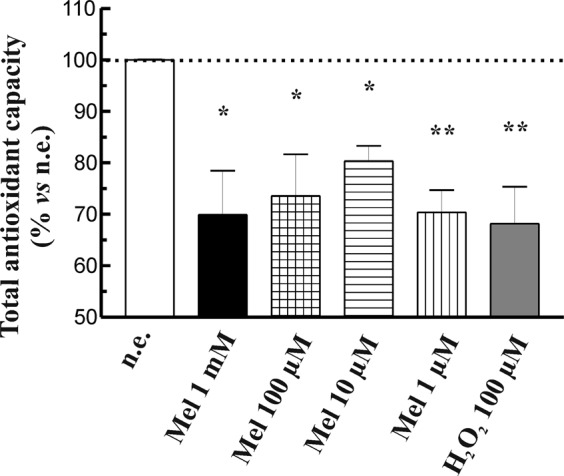
Effect of melatonin on total antioxidant capacity. PSC were incubated with melatonin (1 mM, 100 µM, 10 µM or 1 µM) and then TAC was determined. H_2_O_2_ (100 µM) was used as a control of oxidation. Values show the mean ± S.E.M. of normalized values expressed as % with respect to non-stimulated cells. A horizontal dotted line represents the value observed in non-stimulated cells. Data are representative of three independent experiments (n.e., non-stimulated cells; Mel, melatonin; H_2_O_2_, hydrogen peroxide; *P < 0.05; and **P < 0.01 *vs* non-stimulated cells).

We additionally evaluated the TAC of PSC. As shown in Fig. 8, the TAC of cells incubated in the presence of melatonin was decreased in comparison with that noted in non-stimulated cells (incubated in the absence of melatonin). The effect did not depend on the concentration of melatonin used. Incubation of PSC with the oxidant H_2_O_2_ (100 µM) evoked a statistically significant decrease in TAC compared with non-stimulated cells. These results confirm that melatonin induces changes in the oxidative state of PSC.

## Discussion

It is well known that tumors undergo adaptive responses that lead to resistance and accelerated repopulation. This allows them to overcome doses of radiation and chemotherapy. Resistance can occur following different adaptive responses, which are due to the nature of the tumor cells or to the release of factors by immune cells as well as to participation of other cell types present in the tumor microenvironment^9^. In this line a major contributing factor is the characteristic extensive stromal or fibrotic reaction found in tumors^2^.

In some cancer cells, melatonin itself induces apoptosis^10,13,14^ or aids sensitizing cancer cells to therapy^38–41^. In addition, previous results of our laboratory showed that melatonin modulates viability of PSC. This is of relevance because PSC have been pointed out as major players in stromal formation within tumors^17,18^. Therefore melatonin is emerging as a potential tool in the treatment of cancer.

In this study, we provide further evidences that support a potential role for melatonin in the regulation of PSC proliferation by setting-up a prooxidant environment within the cells, which decreases their viability. The oxidative conditions that we have observed might be based on ROS production together with a decrease in TAC of the cells. The latter might have a basis on a reduction of glutathione levels and a decrease in SOD activity. As a whole, the results that we have obtained can be considered relevant bearing in mind that PSC play major roles in fibrosis developed in pancreatic diseases.

The drop in PSC viability that we have observed confirms previous studies of our laboratory^17,18^. Interestingly, a decrease in the proliferation of this cellular type would be a helpful maneuver that could help in diminishing the fibrosis present in the pancreas under pathological conditions, especially in tumors.

Maintenance of adequate cellular red-ox equilibrium is critical for cell function and viability^42^. Conversely to the protective role of melatonin against oxidative stress^43^ the compound can also exhibit prooxidant effects, which have been related with a cytotoxic effect^28^. The analysis of the results that we have obtained showed that melatonin induced ROS production. The generation of ROS could be detected in both the cytosol and the mitochondria, but the contribution of Ca^2+^ was negligible. Participation of mitochondria in ROS generation has been demonstrated^44,45^. These results are in agreement with previous findings of our laboratory, which showed that ROS production was increased in PSC treated with melatonin^17,18^, and confirm the hypothesis of putative prooxidant actions of melatonin in this cellular type. The present research was conducted in order to further investigate other possible points of action of melatonin to exert its prooxidant effects that could explain its actions on PSC viability.

Our results additionally show that melatonin treatment might be accompanied by oxidation of certain cellular structures. This could be reflected by the increase in the oxidation of cellular proteins that we have noted; however, we could not detect changes in the oxidation of lipids (TBARS). From these observations we could assume that melatonin might differentially affect lipids and proteins within the cell. Besides, it could be possible that certain proteins are more prone to oxidation that lipids upon melatonin treatment. Therefore, melatonin effects on protein redox state could lead to the modulation of metabolic pathways regulated by such proteins, which are activated/inactivated due to changes in their oxidative state.

In addition, impairment of mitochondria leads to ROS generation^22,46^. Our results also show that ψ_m_ decreased in the presence of melatonin. At this point we could hypothesize that melatonin might affect mitochondrial physiology in PSC. In fact, different studies have suggested that melatonin alters mitochondrial physiology which is related with cell death^9,14^. Moreover, a few studies using cultured cells found that melatonin stimulated ROS generation at pharmacological concentrations (micro-molar to milli-molar range) in several tumor and non-tumor cells; thus, melatonin functioned as a conditional pro-oxidant^47^.

Additional evidences for a disruption by melatonin of the redox balance in PSC derives from the experiments directed to analyze its effect on glutathione. The glutathione system is a major tool used in the defense against damage caused by ROS. A defeat of antioxidant systems, like a decrease in the GSH content, can lead cells to fault in the control ROS production and, therefore, can induce cell damage and death^48^. Our results show that, in the presence of melatonin the ratio GSH/GSSG decreased. This action depended on the concentration of melatonin used. Higher effects were found at 100 µM and 1 mM of the indole, whereas no detectable changes were noted in cells treated with 1 µM melatonin. The decline in GSH/GSSG ratio that we have noted points towards an increase in oxidized glutathione. This observation might reflect a pro-oxidant action of melatonin. In other words, the decrease in the availability of reduced glutathione could be related with the increase in ROS generation evoked by melatonin. These results are in agreement with previous observations of our laboratory, obtained in human PSC, in which we showed that melatonin evoked concentration-dependent changes in glutathione oxidation^18^. Interestingly, it could be feasible that melatonin might exert the same effects in human cells as those noted in murine cells, thus providing putative beneficial actions of the compound on human health as expected from the results obtained in studies carried out on animal cells.

In another set of experiments we have detected an increase in the expression of the Nrf2-regulated antioxidant enzymes GClc, CAT, HO-1 and NQO1. Specifically, GCLc is involved in glutathione synthesis^49^. Nrf2 is required for systemic protection against redox-mediated injury. Under oxidative conditions the Keap1-ARE (antioxidant response element) pathway is activated via the upregulation of Nrf2^50^. Melatonin activates this pathway to induce protective antioxidant actions^24,51^. In our study, the prooxidant conditions evoked by melatonin might activate the Nrf2-regulated pathway in an attempt to counteract the pro-oxidative state that we have observed.

SOD is another enzyme with pivotal role in cellular antioxidant defence^52^. Our results show that SOD activity is decreased in the presence of melatonin. This effect could be explained by a diminished expression of both SOD1 and SOD2, whith a higher effect on SOD1. Our results further suggest that melatonin regulates SOD at the translation level. To our knowledge, this is the first time to show that melatonin decreases the expression of SOD. Findings of other researchers show that melatonin either increases SOD expression^53,54^ or does not induces changes in the levels of these proteins^55^.

Interestinlgy, melatonin exerts prooxidant effects^28^ (Sanchez-Sanchez *et al*., 2011). A SOD activity under a certain level could lead to diminished antioxidant pretection of the cell that, if is not counteracted by other antioxidant defenses, might lead to prooxidant conditions that could compromise cell function and viability. As a consequence, and taking also into account the effects on glutathione that we have mentioned above, the TAC of the cells should be expected to decrease, as we have observed. Therefore, our results point out that melatonin modulates pivotal points of the cellular antioxidant machinery and leads to prooxidant conditions that could drive the mechanisms involved in PSC viability and/or proliferation. In fact, we have shown previously that melatonin induced changes in the phosphorylation state of members of the mitogen-activated protein kinases family, which are involved in cell proliferation and survival. This resulted in a decrease in cell viability^17^.

The concentrations of melatonin that we have employed are not physiological and fairly fall within the pharmacological range^56^. However, pharmacological concentrations of melatonin have been used in a plethora of studies directed to the study of disease^57–59^, including studies carried out our laboratory^14,17,24,60,61^.

In conclusion, we present evidences that stand out melatonin as a compound with the ability to regulate PSC physiology. Despite the protective role that melatonin exerts in a wide variety of cellular types, here we show that the compound induces pro-oxidative conditions that might have consequences on cell viability. It is noteworthy to bear in mind that the actions of melatonin on cellular physiology might be cell- and context-dependent. Contribution of stellate cells to survival and development of transformed epithelia within the pancreas has been documented^62,63^. Thus, strategies directed to controlling the growth of fibrotic tissue within tumors might be challenging in the treatment of cancer^2^. In this line, our results suggest a probable mechanism by which melatonin modulates fibrosis within the pancreas. Therefore, melatonin could be considered a hopeful aid in the therapy of pancreatic cancer.
